# Periodontitis and Metabolic Syndrome: Statistical and Machine Learning Analytics of a Nationwide Study

**DOI:** 10.3390/bioengineering10121384

**Published:** 2023-12-01

**Authors:** Asaf Wilensky, Noa Frank, Gabriel Mizraji, Dorit Tzur, Chen Goldstein, Galit Almoznino

**Affiliations:** 1 Department of Periodontology, Hadassah Medical Center, Faculty of Dental Medicine, Hebrew University of Jerusalem, Jerusalem 91120, Israel; 2 Medical Information Department, General Surgeon Headquarter, Medical Corps, Israel Defense Forces, Tel-Hashomer 02149, Israel; 3 Big Biomedical Data Research Laboratory, Dean’s Office, Hadassah Medical Center, Faculty of Dental Medicine, Hebrew University of Jerusalem, Jerusalem 91120, Israel; 4 Department of Oral Medicine, Sedation & Maxillofacial Imaging, Hadassah Medical Center, Faculty of Dental Medicine, Hebrew University of Jerusalem, Jerusalem 91120, Israel

**Keywords:** big data, machine learning, periodontal medicine, systemic health/disease, dental informatics, electronic medical record, periodontitis, periodontal disease, metabolic syndrome

## Abstract

This study aimed to analyze the associations between periodontitis and metabolic syndrome (MetS) components and related conditions while controlling for sociodemographics, health behaviors, and caries levels among young and middle-aged adults. We analyzed data from the Dental, Oral, and Medical Epidemiological (DOME) record-based cross-sectional study that combines comprehensive sociodemographic, medical, and dental databases of a nationally representative sample of military personnel. The research consisted of 57,496 records of patients, and the prevalence of periodontitis was 9.79% (5630/57,496). The following parameters retained a significant positive association with subsequent periodontitis multivariate analysis (from the highest to the lowest OR (odds ratio)): brushing teeth (OR = 2.985 (2.739–3.257)), obstructive sleep apnea (OSA) (OR = 2.188 (1.545–3.105)), cariogenic diet consumption (OR = 1.652 (1.536–1.776)), non-alcoholic fatty liver disease (NAFLD) (OR = 1.483 (1.171–1.879)), smoking (OR = 1.176 (1.047–1.322)), and age (OR = 1.040 (1.035–1.046)). The following parameters retained a significant negative association (protective effect) with periodontitis in the multivariate analysis (from the highest to the lowest OR): the mean number of decayed teeth (OR = 0.980 (0.970–0.991)); North America as the birth country compared to native Israelis (OR = 0.775 (0.608–0.988)); urban non-Jewish (OR = 0.442 (0.280–0.698)); and urban Jewish (OR = 0.395 (0.251–0.620)) compared to the rural locality of residence. Feature importance analysis using the eXtreme Gradient Boosting (XGBoost) machine learning algorithm with periodontitis as the target variable ranked obesity, OSA, and NAFLD as the most important systemic conditions in the model. We identified a profile of the “patient vulnerable to periodontitis” characterized by older age, rural residency, smoking, brushing teeth, cariogenic diet, comorbidities of obesity, OSA and NAFLD, and fewer untreated decayed teeth. North American-born individuals had a lower prevalence of periodontitis than native Israelis. This study emphasizes the holistic view of the MetS cluster and explores less-investigated MetS-related conditions in the context of periodontitis. A comprehensive assessment of disease risk factors is crucial to target high-risk populations for periodontitis and MetS.

## 1. Introduction

Periodontitis is a multifactorial chronic inflammatory disease induced by dysbiotic dental biofilm [1]. Despite advances in understanding and treatment, global periodontitis prevalence is increasing [2]. From 1990 to 2019, the age-standardized prevalence rate of severe periodontitis increased by 8.44% worldwide, and in 2019, there were 1.1 billion (95% uncertainty interval: 0.8–1.4 billion) prevalent cases of severe periodontitis globally [2]. Periodontitis is the primary cause of tooth loss in adults worldwide, adversely impacting mastication, nutrition, appearance, and life quality [3]. Azzolino et al. reviewed oral health determinants of malnutrition, describing that a diet poor in micronutrients may lead to a greater inflammatory response of periodontal tissues, and at the same time, the loss of dental elements due to periodontitis can negatively affect the nutritional status of the patient, resulting in discomfort during chewing and leading to a selection of soft and easy-to-chew foods [4]. Therefore, these processes can further exacerbate sarcopenia and frailty [4]. Moreover, with increasing age, people may experience physical and cognitive decline, which may result in poor oral hygiene, leading to an increased incidence of periodontitis [4].

Periodontitis is a focus in the “periodontal medicine” field, linked to around 50 systemic diseases, including metabolic syndrome (MetS) [5,6]. MetS represents a cluster of cardiometabolic risk factors that can co-occur in an individual, including elevated plasma glucose, central obesity, dyslipidemia, and hypertension [7]. MetS has been linked to obesity-related disorders such as non-alcoholic fatty liver disease (NAFLD) [8] and obstructive sleep apnea (OSA) [9]. MetS poses a significant public health burden, with a global prevalence of approximately 20–25% [7]. Multiple definitions of MetS exist, all agreeing on the defining components but differing in the suggested diagnostic criteria [10].

Both MetS and periodontitis result from multifactorial causes linked to immune and inflammatory responses [11], and a bidirectional relationship exists between them [12].

The underlying mechanisms linking periodontitis to MetS include inflammatory mechanisms where proinflammatory cytokines originating from the gingiva infiltrate the bloodstream and increase oxidative stress, which may facilitate insulin resistance and atherosclerotic changes, and both may lead to MetS development [13]. The connection is bidirectional, as inflammatory cytokines resulting from MetS components may increase the oxidative stress in the gingiva [13]. Elevated blood glucose induces various proinflammatory effects impacting multiple bodily systems, particularly the periodontal tissues [12]. Adipokines from adipose tissue, such as TNF-α, IL-6, and leptin, contribute to inflammation. The hyperglycemic state leads to the deposition of advanced glycation end products (AGEs) in periodontal tissues, triggering local cytokine release and altered inflammatory responses via the receptor for AGE (RAGE) [12]. Diabetic conditions also modify neutrophil function, intensifying the respiratory burst and delaying apoptosis, thereby increasing periodontal tissue destruction [12]. Local cytokine production in periodontal tissues may reciprocally influence glycemic control through systemic exposure, impacting insulin signaling [12]. These factors collectively contribute to dysregulated inflammatory responses in periodontal tissues, exacerbated by the chronic bacterial challenge in the subgingival biofilm and further compounded by smoking [12,13].

Evidence for these mechanisms was revealed, for example, by Ghorbani et al., who demonstrated that the combination of ischemic heart disease and periodontitis is associated with a lower activity of Paraoxonase-1 (PON-1), a new biomarker representing both anti atherosclerotic and antioxidant activity [14]. Narendran et al. assessed the myocardial strain among controlled hypertensive patients with periodontitis and showed that an increase in the periodontal inflamed surface area (PISA) score may cause mild alterations in the global longitudinal strain (GLS) score, which could indicate the possible influence of periodontitis on myocardial activity [15]. Moreover, homocysteine was suggested as a marker of inflammation in patients with periodontitis, and Khudan et al. demonstrated in rats that chronic hyperhomocysteinemia enhances disturbances in bone metabolism in lipopolysaccharide (LPS)-induced periodontitis [16].

However, the literature is conflicting regarding the associations between MetS and periodontitis, with some studies reporting a positive association [6,17,18], while other studies reporting conflicting or null results [19,20]. A systematic review with meta-analysis concluded that the study effect size was influenced by the year of publication, study design, and MetS diagnostic criteria, contributing to inter-study variability [20]. Prior contradictory results may stem from overlooking key confounders in the common risk factor model, such as age, socioeconomic status, smoking, obesity, nutrition, and hygiene [21].

Due to the demand for comprehensive data, interest has grown in using electronic medical records (EMRs) for big data analysis to study dental–systemic interactions using a machine learning (ML) approach [22]. The use of artificial intelligence (AI) technology in clinical practice is an emerging and debated topic, both for its possible diagnostic and therapeutic implications [23]. Through ML, it is possible to exploit multiple variables using easy and rapid extraction, such as the clinical history of the patients as well as anthropometric or demographic characteristics, to facilitate the identification of otherwise complex pathologies [23]. In dentistry, several studies applied ML for periodontitis classification, producing encouraging results [24,25,26,27,28]. Limitations of these studies include using data from a single institution [25], not consistently including social determinants of health and systemic conditions or relying on patient-reported data, and not involving EMRs. Patel et al. stressed that analyzing common periodontitis risk factors (e.g., periodontal pockets) lacks clinical utility, given clinician awareness. They suggested focusing on the underlying risk factors as targets for preventive interventions [25]. Furthermore, prior studies employed ML or statistics; however, combining both enhances comparisons and validates findings.

The rationale of this study involves the use of a novel methodology combining statistical and ML approaches in artificial intelligence technology to study the association between periodontitis and MetS, by utilizing a big data repository of a nationally representative population of young-to-middle-aged adults. This comprehensive repository allowed us to examine parameters from different facets of life, namely sociodemographics, health-related habits, medical history, and dental history, and thus consider the presence of numerous confounding parameters that had been gathered using a strict protocol for dental and medical disease definitions. This study aimed to address the unmet needs by analyzing the associations between periodontitis and MetS components and related conditions among young-to-middle-aged adults while controlling for sociodemographics, health behaviors, and caries levels. Our hypothesis suggests a positive association between periodontitis and certain MetS-related conditions. By addressing these research objectives, our aim is to advance periodontal medicine research and illuminate potential avenues for future clinical applications.

## 2. Methods

### 2.1. Data Source

This cross-sectional study is a part of the record-based nationwide Dental, Oral, and Medical Epidemiological (DOME) big data study [22,29,30,31]. These earlier articles featured and detailed the DOME study, with one dedicated to its protocol and methods [29]. Briefly, to achieve the objectives, we utilized the DOME study’s database, a large-scale, structured, and comprehensive repository that integrates sociodemographic, medical, and dental databases from a nationally representative population of young-to-middle-aged adults of military personnel within the Israel Defense Forces (IDF). Instead of relying solely on patient-provided information, this study cross-referenced data from three electronic databases: (1) dental patient records (DPRs), (2) medical records (i.e., computerized patient records (CPRs)), and (3) sociodemographic records. Data extraction was performed using the IDF Medical Information Department and was completely anonymous [29]. As detailed in “DOME Protocol and Study Methods”, the data warehouse (DWH) of the IDF Medical Corps combines information from several operational source systems into one comprehensive database. The collected data are classified in the DWH into Oracle database schema according to the data world of the original operational sources (e.g., CPR schema and DPR schema). Data management from the DWH was performed using Statistical Analysis System (SAS) version 7.1.

### 2.2. Ethical Clearance

This study was approved by the Medical Corps Institutional Review Board, with approval number IDF-1281-2013, and conformed to the guidelines of the STROBE (Strengthening the Reporting of Observational Studies in Epidemiology). This study was granted informed consent exemption as it involved the retrospective analysis of anonymous electronic records.

### 2.3. Study Eligibility Criteria

***Inclusion criteria***: Men and women, aged 18–50 years, who visited the IDF dental clinics between 1 January 2015 and 1 January 2016, whose information is recorded in the sociodemographic, medical, and dental military electronic records, and who had periodontal status examinations recorded in the dental records were included in this study.

***Exclusion criteria*:** Subjects lacking this information in these databases were not included.

### 2.4. Variables’ Definitions

The definitions of variables are detailed in “DOME Protocol and Study Methods” [29], and below, we provide a concise overview:

#### 2.4.1. The Dependent Variable: Periodontitis

As described in our previous publications [29,30,31], periodontitis was defined according to the American Academy of Periodontology guidelines [32] (data collected before the new classification was published). Furthermore, due to possible pseudo pockets, the assessment of radiographic bone loss was deemed essential, as defined by crest cement junction distance exceeding 2 mm in more than one tooth, without observable causes (e.g., faulty restorations or overhangs, interproximal cavitation, etc.) [29].

#### 2.4.2. Independent Variables

##### Sociodemographic Variables

1. Age in years;
2. Sex (men/women);
3. *Education*: Educational attainment categorized as high school and below, technical college, or academic;
4. *Locality of Residence*: Classification into urban Jewish, urban non-Jewish, or rural areas;
5. *Socioeconomic Status (SES)*: Socioeconomic status as derived from the Israeli Ministry of the Interior records, categorized as low (1st–4th), medium (5th–7th), or high (8th–10th) deciles;
6. *Birth countries:* North America, Eastern Europe, Western Europe, Ethiopia, Africa, Asia, South America, and Israel.

All sociodemograhic variables are listed in Table 1.

##### Health Behaviors

The following self-reported health behaviors were included (yes/no): tooth brushing (at least once a day), current smoking status, consumption of cariogenic diet (snacks and/or sweets food intake between/instead of meals), and consumption of sweetened beverages (above one glass per day).

##### Definition of Medical Diagnoses and Auxiliary Test Results

The CPR database uses the International Classification of Diseases, 9th Revision, Clinical Modification (ICD-9-CM) as the basis for diagnosis. The extracted diagnoses and auxiliary test results as part of the evaluation of MetS components are displayed in Table 2.

### 2.5. Analytical Approach

A novel integrated method using statistical and ML models was employed for data analysis.

#### 2.5.1. Statistical Analyses

The IBM (International Business Machines) SPSS (Statistical Package for the Social Sciences) software version 28.0 (Chicago, Illinois, United States) was used to conduct the statistical analyses.

***Descriptive statistics*:** Continuous variables are displayed as means and standard deviations. Categorical variables are displayed as frequencies and percentages.

***Univariate analysis***: The associations between periodontitis and the independent variables were analyzed with Pearson’s chi-square (χ^2^) or likelihood ratio test for categorical parameters and a non-paired *t*-test for continuous variables. To calculate the odds ratio (OR), we employed binary logistic regression analysis for binary categorical dependent variables and linear regression analysis for continuous dependent variables.

***False discovery rate (FDR) procedure*:** Controlling type I errors in multiple hypothesis testing is of paramount importance in biomedical research. Therefore, following the univariate analysis, we applied the Benjamini–Hochberg (BH) procedure, which provides a balance between controlling the FDR and maintaining statistical power.

***Analysis of multicollinearity***: A linear regression model that included collinearity tests was conducted with independent variables that retained statistical significance following the BH procedure. Only one of the highly correlated variables was selected based on the context. Variance inflation factors (VIFs), typically indicating collinearity at VIF > 10, were set at VIF 2.5 as a limit, due to the potential issues in weaker models.

***Multivariate analysis***: A multivariate binary logistic regression analysis was performed, including independent variables that were statistically significant following the BH procedure and were not collinear.

#### 2.5.2. Machine Learning (ML) Models

The Python scikit-learn package [33] was used to run ML models.

***XGBoost ML algorithm*:** We utilized eXtreme Gradient Boosting (XGBoost), a powerful gradient-boosting framework for supervised learning problems that can be used for both regression and classification applications [34,35]. XGBoost iteratively trains decision trees on the residuals of a previous iteration, where the residuals are the differences between the actual and predicted values [34,35]. The algorithm also employs regularization techniques to prevent overfitting and improve model generalization [34,35]. We used the XGBoost algorithm to generate a list of prioritized variables according to their importance in the task of periodontitis classification [35]. We varied the ratios of training and testing (e.g., 70–30% and 80–20%) and conducted five-fold cross-validation [36].

*Sensitivity analysis*: Two additional ML algorithms were used to determine the features’ importance, to confirm the validity of the XGBoost ML model: Gini importance [37] and information gain [38].

**Gini Importance ML algorithm**: Gini importance is a technique for determining the importance of input features in a random forest model [37,39]. It calculates the overall reduction in the Gini impurity index to which each feature contributes across all decision trees in the forest. The Gini impurity index measures the homogeneity of the target variable within a decision tree node [37,39].

**Information gain ML algorithm**: Information gain (using entropy) [38] is a feature selection method commonly used in ML that measures the entropy or uncertainty reduction of a given dataset when features are included. This algorithm calculates the information gain for each feature by comparing the entropy of the original dataset with the entropy of the dataset after the feature is added. Features with high information gain are considered more informative and are selected for use in the final model [38].

*Compliance with Reporting Guidelines in Machine Learning Research*: The adherence of the study to reporting guidelines was assessed using the TRIPOD (Transparent Reporting of a Multivariable Prediction Model for Individual Prognosis or Diagnosis) checklist, (www.tripod-statement.org, accessed on 1 November 2023). This checklist comprises 20 main items and 31 subitems, covering various aspects of a prediction model’s validation, such as title, abstract, methods, results, and funding disclosure. Each item received a binary rating of “1” for adherence or “0” for non-adherence. Subsequent scrutiny unveiled that this research rigorously adheres to all the elements prescribed by the TRIPOD, with three items deemed irrelevant. Detailed documentation of compliance with each TRIPOD item is provided.

## 3. Results

This research consisted of 57,496 records of patients, and the prevalence of periodontitis in the study population was 9.79% (5630/57,496). Table 1 presents the associations of periodontitis with sociodemographics, health behaviors, and the mean number of untreated decayed teeth. The following parameters were positively associated with periodontitis:

1. Technical (odds ratios (OR) and 95% confidence interval (CI) = 2.035 (1.107–1.317)) and academic education (OR = 1.208 (1.107–1.317)) compared to high school education;
2. High (OR = 1.277 (1.102–1.480)) and medium (OR = 1.254 (1.084–1.452)) SES compared to low SES;
3. Rural (OR = 2.017 (1.479–2.751)) and urban non-Jewish (OR = 1.117 (1.032–1.210)) compared to urban Jewish localities;
4. African birth country (OR = 1.648 (1.066–2.546)) compared to native Israelis;
5. Current smoker status (OR = 1.682 (1.531–1.849));
6. Brushing teeth at least once a day (OR = 3.182 (2.940–3.443));
7. Cariogenic diet consumption (OR = 1.966 (1.860–2.078));
8. Sweetened beverage consumption (OR = 1.632 (1.544–1.725));
9. Age (OR = 1.035 (1.032–1.039)).

Periodontitis was negatively associated with the mean number of untreated decayed teeth (OR = 0.972 (0.961–0.982)) and with North America as the birth country (OR = 0.715 (0.596–0.898)). There were no statistically significant associations between periodontitis and sex (OR = 1.028 (0.966–1.095)) (Table 1).

**Table 1:** The associations of periodontitis with sociodemographic parameters, health behaviors, and the mean number of untreated decayed teeth; Pearson’s chi-square *; likelihood ratio ^; non-paired *t*-test **; generalized linear models ^^; binary logistic regression #.

Parameter	Variable	Periodontitis No. (%)	Without Periodontitis No. (%)	*p* Value	OR (95% Confidence Interval) #
Sex	Men	4190 (74.4)	38,322 (73.9)	0.384 *	1.028 (0.966–1.095)
	Women	1440 (25.6)	13,544 (26.1)		1
Educational level	High school	4306 (76.6)	43,111 (83.2)	<0.001 ^	1
	Technicians	664 (11.8)	3267 (6.3)		2.035 (1.107–1.317)
	Academic	653 (11.6)	5414 (10.5)		1.208 (1.107–1.317)
Socioeconomic status (SES)	Low	209 (3.8)	2414 (4.7)	0.005 ^	1
	Medium	3017 (54.3)	27,785 (54.2)		1.254 (1.084–1.452)
	High	2333 (42.0)	21, 098 (41.1)		1.277 (1.102–1.480)
Residency location	Urban Jewish	4772 (85.0)	44,792 (86.7)	<0.001 ^	1
	Urban non-Jewish	790 (14.1)	6637 (12.8)		1.117 (1.032–1.210)
	Rural	49 (0.9)	228 (0.4)		2.017 (1.479–2.751)
Birth country	Western Europe	494 (8.8)	4293 (8.3)	0.006 ^	1.066 (0.976–1.176)
	Eastern Europe	81 (1.4)	738 (1.4)		1.017 (0.807–1.282)
	Asia	29 (0.5)	199 (0.4)		1.351 (0.914–1.966)
	Ethiopia	104 (1.8)	884 (1.7)		1.090 (0.888–1.399)
	Africa	24 (0.4)	135 (0.3)		1.648 (1.066–2.546)
	North America	81 (1.4)	1050 (2.0)		0.715 (0.596–0.898)
	South America	49 (0.9)	378 (0.7)		1.201 (0.891–1.620)
	Israel	4767 (84.7)	44,178 (85. 2)		1
Current smoker status	No	5071 (90.1)	48,676 (93.8)	<0.001 *	1
	Yes	559 (9.9)	3190 (6.2)		1.682 (1.531–1.849)
Brushing teeth once a day or more	No	752 (13.4)	17,068 (32.9)	<0.001 *	1
	Yes	4878 (86.6)	34,798 (67.1)		3.182 (2.940–3.443)
Cariogenic diet consumption	No	2534 (45.0)	31,987 (67.1)	<0.001 *	1
	Yes	3096 (55.0)	19,879 (38.3)		1.966 (1.860–2.078)
Sweetened drink consumption	No	2165 (46.4)	30,394 (58.6)	<0.001 *	1
	Yes	3015 (53.6)	21,472 (41.4)		1.632 (1.544–1.725)
Parameter		Mean ± SD		*p* value	OR (95% Confidence Interval) ^^
Age	Without periodontitis	22.4 ± 6.5		<0.001 **	1
	Periodontitis	24.3 ± 8.3			1.035 (1.032–1.039)
Mean number of untreated decayed teeth	Without periodontitis	2.22 ± 2.85		<0.001**	1
	Periodontitis	2.00 ± 2.75			0.972 (0.961–0.982)

**Figure 1:**
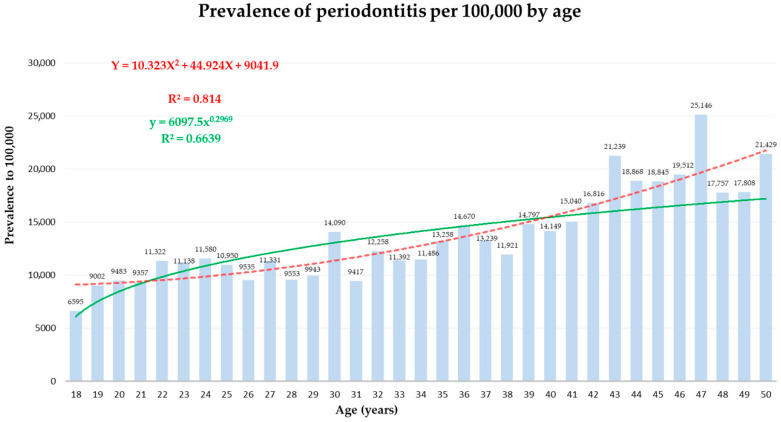
Prevalence of periodontitis per 100,000 by age among the study population.

Figure 1 presents the prevalence of periodontitis per 100,000 by age among the study population, demonstrating that periodontitis prevalence increases with age.

Table 2 presents the associations of periodontitis with MetS-related diagnoses and with auxiliary tests. Periodontitis was positively associated with the following medical diagnoses (from highest to lowest OR): obstructive sleep apnea (OSA) (OR = 3.477 (2.517–4.803)); non-alcoholic fatty liver disease (NAFLD) (OR = 2.448 (1.991–3.008)); diabetes type 2 (OR = 2.199 (1.549–3.121)); obesity (OR = 1.629 (1.486–1.787)); hyperlipidemia (OR = 1.518 (1.181–1.950)); cardiac disease (OR = 1.490 (1.300–1.707)); and hypertension (OR = 1.413 (1.222–1.634)) (Table 2). Periodontitis had statistically significantly higher test values in most auxiliary tests, but the associations were weak, with ORs close to 1 (Table 2).

**Table 2:** The associations between periodontitis with metabolic syndrome components, consequences, and related conditions; Pearson’s chi-square *; binary logistic regression **, non-paired *t*-test ^; generalized linear models ^^.

Parameter	Variable	Periodontitis No.% (%)	Without Periodontitis No. (%)	*p* Value *	OR (95% Confidence Interval) **
Hypertension	No	5413 (96.1)	50,435 (97.2)	<0.001	1
	Yes	217 (3.9)	1431 (2.8)		1.413 (1.222–1.634)
Diabetes type 2	No	5591 (99.3)	51,702 (99.7)	<0.001	1
	Yes	39 (0.7)	164 (0.3)		2.199 (1.549–3.121)
Hyperlipidemia	No	5558 (98.7)	51,427 (99.2)	0.001	1
	Yes	72 (1. 3)	439 (0.8)		1.518 (1.181–1.950)
Obesity	No	5044 (89.6)	48,414 (93.3)	<0.001	1
	Yes	586 (10.4)	3452 (6.7)		1.629 (1.486–1.787)
Cardiac disease	No	5380 (95.6)	50,297 (97.0)	<0.001	1
	Yes	250 (4.4)	1569 (3.0)		1.490 (1.300–1.707)
Obstructive sleep apnea (OSA)	No	5579 (99.1)	51,730 (99.7)	<0.001	1
	Yes	51 (0.9)	136 (0.3)		3.477 (2.517–4.803)
Non-alcoholic fatty liver disease (NAFLD)	No	5514 (97.9)	51,424 (99.1)	<0.001	1
	Yes	116 (2.1)	442 (0.9)		2.448 (1.991–3.008)
Parameter	N	Study group	Mean ± SD	*p* value ^	OR (95% confidence interval) ^^
Body mass index (BMI) kg/m2	24,596	Without periodontitis	24.36 ± 4.41	0.00009	1
	2880	Periodontitis	24.70 ± 4.39		1.017 (1.009–1.026)
Cholesterol (mg/dL)	11,481	Without periodontitis	176.65 ± 35.73	0.012	1
	1646	Periodontitis	179.02 ± 36.53		1.002 (1.001–1.003)
High-density lipoprotein (HDL) (mg/dL)	11,481	Without periodontitis	47.95 ± 11.62	0.006	1
	1646	Periodontitis	47.11 ± 11.24		0.994 (0.989–0.998)
Low-density lipoprotein (LDL)	7479	Without periodontitis	108.92 ± 30.70	0.048	1
	1106	Periodontitis	110.87 ± 30.92		1.002 (1.000–1.004)
Non-HDL cholesterol (mg/dL)	6842	Without periodontitis	130.77 ± 35.05	0.007	1
	1103	Periodontitis	133.79 ± 35.56		1.002 (1.001–1.004)
Triglycerides (mg/dL)	11,484	Without periodontitis	106.47 ± 64.74	0.017	1
	1646	Periodontitis	110.55 ± 67.45		1.001 (1.000–1.002)
Very low-density lipoprotein (VLDL) (mg/dL)	11,461	Without periodontitis	20.96 ± 11.30	0.013	1
	1644	Periodontitis	21.71 ± 11.90		1.006 (1.001–1.010)
Glycated hemoglobin (HbA1c) (%)	847	Without periodontitis	5.42 ± 0.98	0.63	1
	158	Periodontitis	5.47 ± 1.11		1.040 (0.884–1.223)
Oral glucose tolerance test-T0 (mg/dL)	312	Without periodontitis	89.90 ± 20.12	0.017	1
	51	Periodontitis	97.90 ± 31.18		1.012 (1.001–1.023)
Oral glucose tolerance test-T60 (mg/dL)	438	Without periodontitis	133.02 ± 44.13	0.008	1
	60	Periodontitis	151.70 ± 87.62		1.005 (1.001–1.010)
Oral glucose tolerance test-T120 (mg/dL)	119	Without periodontitis	105.24 ± 38.21	0.040	1
	23	Periodontitis	123.39 ± 45.01		1.010 (1.000–1.010)

We performed the BH procedure to decrease the FDR (see Table 3). Only the independent variables that were statistically significant following the BH procedure entered the next step of collinearity statistics.

The results of collinearity statistics shown in Table 4 ruled outcollinearity (VIF < 2.5). Subsequently, independent variables that were statistically significant following the BH procedure and were not collinear were used for the multivariate binary logistic regression analysis (Table 4). The following parameters retained a significant positive association with periodontitis in the multivariate analysis, the results of which are shown in Table 4 (from the highest to the lowest OR): brushing teeth (OR = 2.985 (2.739–3.257)); OSA (OR = 2.188 (1.545–3.105)); consumption of cariogenic diet (OR = 1.652 (1.536–1.776)); NAFLD (OR = 1.483 (1.171–1.879)); smoking (OR = 1.176 (1.047–1.322)); and age (OR = 1.040 (1.035–1.046)) (Table 4).

The following parameters retained a significant negative association with periodontitis in the subsequent multivariate analysis (from the highest to the lowest OR): the mean number of untreated decayed teeth (OR = 0.980 (0.970–0.991)); North America as the birth country (OR = 0.775 (0.608–0.988)) compared to native Israelis; urban non-Jewish (OR = 0.442 (0.280–0.698)) and urban Jewish (OR = 0.395 (0.251–0.620)) compared to the rural locality of residence (Table 4).

**Figure 2:**
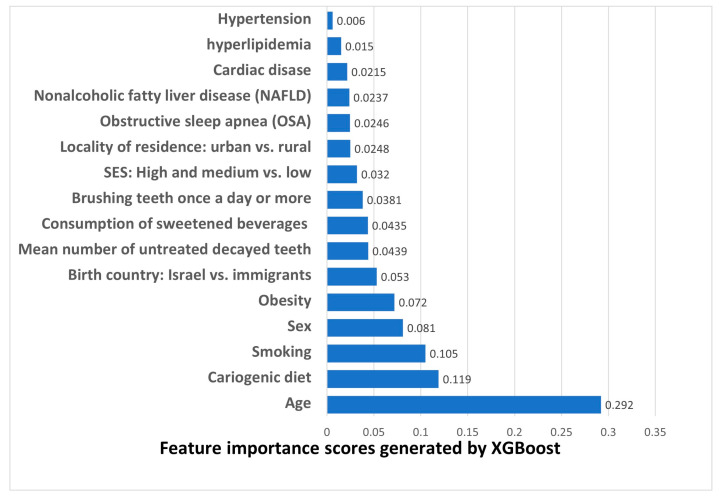
The ranking chart for clinical features’ importance generated using the XGBoost machine learning algorithm for periodontitis set as the target variable. Five-fold cross-validation; train/test 80%/20%.

In the ensuing stage, we employed ML algorithms for the task of periodontitis diagnosis classification. The Gini importance and information gain algorithms resulted in outcomes akin to the performance metrics of the XGBoost model. Consequently, we report the results attained using the XGBoost algorithm in Figure 2. The model yielded an area under the curve (AUC) = 0.63, an accuracy = 0.65, precision = 0.19, an F1 score of 0.275, and a recall score of 0.506. Cut-offs for AUC discrimination results of 0.7 ≥ AUC ≥ 0.6 are considered acceptable discrimination [40]. However, with a precision rate nearly double the periodontitis prevalence (19% vs. 9.79%), the XGBoost model excels in accurate disease detection while minimizing false positives. The model ranked the significance of the features in relation to the target variable (periodontitis) as follows: age was ranked first, followed by cariogenic diet (second), and smoking (third). The top-ranked MetS-related conditions were obesity (fifth), followed by OSA (twelfth), and NAFLD (thirteenth).

## 4. Discussion

In this research, we analyzed the associations of periodontitis with MetS among a nationwide population of 57,496 young and middle-aged adults, utilizing the DOME comprehensive repository. This provides us the unique opportunity to cross-check sociodemographic, dental, and medical parameters against periodontitis diagnosis and simultaneously analyze important confounders and mediators on an unmatched scale. In line with the current periodontitis classification, the collected data encompassed age, smoking habits, and medical comorbidities. The overall results obtained using our novel method combining statistical and ML approaches allowed us to establish a profile of the “patient vulnerable to periodontitis” that includes older age, rural locality, smoking, brushing teeth, cariogenic diet consumption, obesity, OSA, NAFLD, and having fewer untreated decayed teeth. Individuals born in North America had a lower prevalence of periodontitis than native Israelis.

Periodontitis prevalence: Among the nationwide sample of the Israeli population aged 18–50 years, periodontitis prevalence was 9.79%. According to the Global Burden Study, the prevalence of periodontitis increased from 1990 to 2019 [2], affecting almost 50%, and its severe form affects 9.8% (1990–2017) [41]. Our lower prevalence compared to the literature may result from our focus on young-to-middle-aged adults and the inclusion of radiographic bone loss in the periodontitis definition.

Sociodemographic parameters: For the task of periodontitis classification, age was ranked first in the ML model (Figure 2) and retained a significant association in the statistical multivariate analysis (Table 4). In agreement, previous studies demonstrated that the risk of periodontitis rises with age globally, with a notable increase between the third and fourth decades of life [2]. Greater periodontal destruction in the elderly reflects lifetime disease accumulation [11], and thus age is recognized as a risk factor for the future progression of alveolar bone loss [42]. A geoscience approach links periodontitis and MetS as phenotypic expressions of accelerated biological aging [43].

Our study, like prior research [44], revealed a positive association between higher education and periodontitis [Table 1], attributed to multicollinearity between age and education, which prompted the exclusion of education from the multivariate analysis.

Sex was ranked fourth in the ML feature importance (Figure 2), although the association between sex and periodontitis did not reach statistical significance (Table 1). While systematic reviews provide evidence for a lower prevalence of periodontitis among women, men do not appear to have a higher risk for rapid periodontal destruction than women [45]. Previous studies related sex-based periodontal differences to oral care behaviors of men rather than genetics [46], underscoring our holistic analysis.

Consistent with prior investigations [2], periodontitis was more prevalent among those with low and medium SES (Table 1). However, SES did not retain a statistically significant association with periodontitis in a multivariate analysis (Table 4) and ranked tenth in the ML feature importance model (Figure 2), highlighting the significance of other factors.

Rural locality retained statistical significance even following multivariate analysis (Table 4) and ranked eleventh in the ML feature importance model (Figure 2), consistent with prior research [47]. Since military dental clinics are spread throughout the country, we attribute our findings to the consequences of living in a rural area, not clinic proximity.

An important socioeconomic determinant is the birth country, particularly in Israel, which is known as an immigrant state. North American-born individuals had a lower prevalence of periodontitis than native Israelis (Table 4), and the birth country parameter (Israeli natives vs. immigrants) was ranked sixth in the ML feature importance model (Figure 2). This is in line with the Global Burden of Severe Periodontitis Study, 1990–2019 [2], which demonstrated less prevalent cases of periodontitis in high-income North America and Canada compared to Israel [2].

Supporting our holistic approach to identifying novel sociodemographic parameters as predictors of periodontitis, Alqahtani1 et al. recently published a cross-sectional study of the 2013–2014 National Health and Nutrition Examination Survey (*n* = 4555) and identified age and education level as the two most important predictors for the presence and severity of periodontitis using ML models [26]. Other significant factors included alcohol use, type of medical insurance, sex, and non-white race [26].

Health behaviors: In the ML model, cariogenic diet consumption ranked second, smoking third, sweetened beverage consumption ranked eighth, and teeth brushing ranked ninth (Figure 2). These parameters were also positively associated with periodontitis in the multivariate analysis (Table 4). Interestingly, patients with periodontitis exhibited better teeth-brushing habits and fewer decayed teeth. This may be due to the instructions provided to them or because they were being observed as part of the study (i.e., the Hawthorne effect). However, information bias is unlikely as patients with periodontitis also reported higher rates of smoking and consumption of cariogenic diet and sweetened beverages, all of which are known as unhealthy habits.

Decayed teeth: Periodontitis patients had fewer decayed teeth (Table 4), and decayed teeth were ranked seventh place in the ML feature importance model (Figure 2). In agreement, Sewon et al. observed a higher prevalence of caries-free teeth and molars in individuals with periodontitis [48]. Conversely, other studies found that periodontitis is more prevalent in the presence of caries [49].

MetS. related conditions: Our primary objective was to analyze the association between periodontitis and MetS-related conditions using statistical and ML models. Interestingly, the only MetS-related conditions that retained statistical significance with periodontitis following the multivariate statistical analysis were NAFLD and OSA (Table 4), and the highest-ranked MetS-related conditions in the ML model were obesity (ranked fifth), followed by OSA (twelfth) and NAFLD (thirteenth) (Figure 2). Periodontitis associations with obesity, NAFLD, and OSA are underexplored compared to diabetes and cardiovascular links, potentially due to the inadequate consideration of concurrent MetS components. NAFLD, the most prevalent chronic liver disease worldwide, involves hepatic fat accumulation and is strongly associated with obesity [8]. Likewise, OSA is marked by recurrent sleep airway obstruction, and therefore patients with OSA may benefit from treatment like palate surgery, reducing not only the apnea and hypopnea indices and daytime sleepiness but also associated with mood comorbidities [50]. OSA is closely tied to obesity [9]. Obesity appears to be a critical factor driving the pathogenesis of both NAFLD and OSA [51]. Indeed, an international expert panel from 22 countries redefined NAFLD as a metabolic dysfunction-associated fatty liver disease (MAFLD) [8]. In our recent publications, we separately studied OSA [31] and NAFLD [30] and found a positive association with periodontitis. Our findings are also in line with other publications linking obesity and periodontitis, including a Mendelian randomization study suggesting a potential causal association between obesity and periodontitis [52]. Wang et al. conducted a systematic comparison of six machine learning algorithms to develop and validate a prediction model to predict heart failure risk in middle-aged and elderly patients with periodontitis, and the variables in the final model were ranked in the descending order of importance as myocardial infarction, age, diabetes, and race. While their research question is different, the study by Wang et al. and the current study both highlight the importance of demographic factors such as age and race, as well as MetS-related conditions, in the context of periodontitis [53]. Overall, our study highlights the importance of considering the whole MetS cluster and sheds light on less investigated MetS-related conditions in the context of periodontitis.

Strengths and limitations

The present study’s strengths include a large sample size and rigorous protocol incorporating dental, medical, and sociodemographic databases, with consistent and strict definitions for all patients. Clinical and radiographic assessments were utilized for dental parameters. Dental and medical indexes were derived from records rather than relying on patient-reported data, except for health behaviors. Furthermore, this study employed a novel analytical approach combining both statistical methods and ML algorithms.

The limitations of this study include its cross-sectional design, preventing the establishment of causality. Another limitation pertains to the new classification for periodontitis, which considers staging including furcation involvement, tooth hypermobility, and the presence of infra-bony defects, parameters that were not measured and analyzed in this study. Although multiple confounding factors were taken into account, there are residual confounding factors that were not analyzed such as genetics, microbiome, and childhood and past exposures. Future research should involve long-term longitudinal population-based epidemiological surveys conducted in different settings and populations that will incorporate multiomics data to increase generalizability, account for causal inferencing, and address these limitations. 

## 5. Conclusions

The prevalence of periodontitis among a nationwide sample of the Israeli population aged 18–50 years was 9.79%. We identified a profile of the “patient vulnerable to periodontitis” characterized by older age; rural residency; smoking; brushing teeth; cariogenic diet; comorbidities of obesity, OSA, and NAFLD; and fewer untreated decayed teeth. North American-born individuals had a lower prevalence of periodontitis than native Israelis. Healthcare authorities should be familiar with the vulnerable patient profile for periodontitis. Assessment should encompass all relevant risk factors, including systemic health, sociodemographic characteristics, and general and dental health-related factors. A comprehensive assessment of disease risk factors is crucial to target high-risk populations for periodontitis and MetS.

## Figures and Tables

**Table 3:** Benjamini–Hochberg (BH) procedure performed to decrease the false discovery rate (FDR).

	Corrected *p* Value	i	*p* Value Level for FDR	Number of Comparisons	Crit	BH Test Result
Glycated hemoglobin (HbA1c)	0.63	28	0.05	28	0.05	Not Significant
Sex	0.384	27	0.05	28	0.048214	Not Significant
Low-density lipoprotein (LDL)	0.048	26	0.05	28	0.046429	Not Significant
Oral glucose tolerance test-120	0.04	25	0.05	28	0.044643	Significant
Oral glucose tolerance test-T0	0.017	24	0.05	28	0.042857	Significant
Triglycerides	0.017	23	0.05	28	0.041071	Significant
Very low-density lipoprotein (VLDL)	0.013	22	0.05	28	0.039286	Significant
Cholesterol	0.012	21	0.05	28	0.0375	Significant
Oral glucose tolerance test-T60	0.008	20	0.05	28	0.035714	Significant
Non-HDL cholesterol	0.007	19	0.05	28	0.033929	Significant
HDL	0.006	18	0.05	28	0.032143	Significant
Birth country	0.006	17	0.05	28	0.030357	Significant
Socioeconomic status (SES)	0.005	16	0.05	28	0.028571	Significant
Body mass index (BMI) kg/m2	0.00009	15	0.05	28	0.026786	Significant
Non-alcoholic fatty liver disease (NAFLD)	0	14	0.05	28	0.025	Significant
Obstructive sleep apnea (OSA)	0	13	0.05	28	0.023214	Significant
Cardiac disease	0	12	0.05	28	0.021429	Significant
Obesity	0	11	0.05	28	0.019643	Significant
Hyperlipidemia	0	10	0.05	28	0.017857	Significant
Diabetes type 2	0	9	0.05	28	0.016071	Significant
Hypertension	0	8	0.05	28	0.014286	Significant
Mean number of untreated decayed teeth	0	7	0.05	28	0.0125	Significant
Cariogenic diet consumption	0	6	0.05	28	0.010714	Significant
Brushing teeth once a day or more	0	5	0.05	28	0.008929	Significant
Current smoker status	0	4	0.05	28	0.007143	Significant
Residency location	0	3	0.05	28	0.005357	Significant
Educational level	0	2	0.05	28	0.003571	Significant
Age	0	1	0.05	28	0.001786	Significant

**Table 4:** Collinearity statistics and multivariate analysis of periodontitis as dependent variable. SE: standard error, VIF: variance inflation factor. Statistically significant *p* values are in bold.

Parameter	Variable	Multivariate Binary Logistic Regression Analysis				Collinearity Statistics Using Linear Regression Analysis	
		B	SE	*p* Value	OR and 95% Confidence Interval	Tolerance	VIF
(Intercept)		0.412	0.837	0.622			
Age		0.040	0.003	**<0.001**	1.040 (1.035–1.046)	0.504	1.986
Residency location—reference rural	Urban Jewish	−0.930	0.230	**<0.001**	0.395 (0.251–0.620)	0.989	1.011
	Urban non-Jewish	−0.816	0.233	**<0.001**	0.442 (0.280–0.698)	0.982	1.019
Socioeconomic status (SES)—reference high	low	−0.146	0.078	0.062	0.864 (0.741–1.007)	0.948	1.055
	Medium	0.008	0.030	0.795	1.008 (0.949–1.070)	0.945	1.058
Birth country: reference Israel	Western Europe	0.051	0.052	0.330	1.052 (0.950–1.166)	0.864	1.158
	Eastern Europe	0.050	0.122	0.685	1.051 (0.827–1.335)	0.959	1.043
	Asia	0.231	0.214	0.279	1.260 (0.829–1.915)	0.843	1.187
	Ethiopia	0.168	0.110	0.127	1.183 (0.954–1.468)	0.971	1.030
	Africa	0.302	0.249	0.225	1.353 (0.830–2.205)	0.835	1.198
	North America	−0.255	0.124	**0.039**	0.775 (0.608–0.988)	0.927	1.078
	South America	0.178	0.163	0.274	1.195 (0.868–1.645)	0.602	1.661
Smoking		0.163	0.059	**0.006**	1.176 (1.047–1.322)	0.756	1.322
Brushing teeth once a day or more		1.095	0.044	**<0.001**	2.985 (2.739–3.257)	0.817	1.224
Consumption of a cariogenic diet		0.502	0.037	**<0.001**	1.652 (1.536–1.776)	0.609	1.643
Consumption of sweetened beverages		0.005	0.037	0.889	1.005 (0.934–1.081)	0.598	1.671
Mean number of untreated decayed teeth		−0.020	0.006	**<0.001**	0.980 (0.970–0.991)	0.798	1.253
Hypertension		0.036	0.084	0.669	1.037 (0.879–1.222)	0.903	1.107
Diabetes type 2		0.243	0.200	0.224	1.275 (0.862–1.886)	0.952	1.050
Hyperlipidemia		0.038	0.140	0.787	1.038 (0.789–1.366)	0.957	1.045
Obesity		0.026	0.062	0.669	1.027 (0.909–1.159)	0/685	1.460
Cardiac disease		0.048	0.078	0.538	1.049 (0.900–1.222)	0.928	1.077
Obstructive sleep apnea (OSA (		0.784	0.178	**<0.001**	2.188 (1.545–3.105)	0.973	1.028
Non-alcoholic fatty liver disease (NAFLD)		0.395	0.121	**0.001**	1.483 (1.171–1.879)	0.905	1.104
Anemia		0.014	0.055	0.802	1.014 (0.910–1.128)	0.886	1. 098

## Data Availability

Data are contained within the article.
